# Supplementary material for: Strategies to Enhance CO2 Electrochemical Reduction from Reactive Carbon Solutions
Source: Molecules. 2023 Feb 18;28(4):1951. doi: 10.3390/molecules28041951 (PMC9960053; doi:10.3390/molecules28041951)
Supplement: Supplementary file 1 [file molecules-28-01951-s001.zip › molecules-2219834-supplementary.pdf]

# Electronic Supporting Information

# Strategies to enhance $CO_2$ electrochemical reduction from reactive carbon solutions

**Carlos Larrea[1], Juan Ramón Avilés-Moreno[1], Pilar Ocón[1]***

[1] Universidad Autónoma de Madrid (UAM), Departamento de Química Física Aplicada, C/Francisco Tomás y Valiente 7, 28049 Madrid, Spain
* Correspondence: pilar.ocon@uam.es

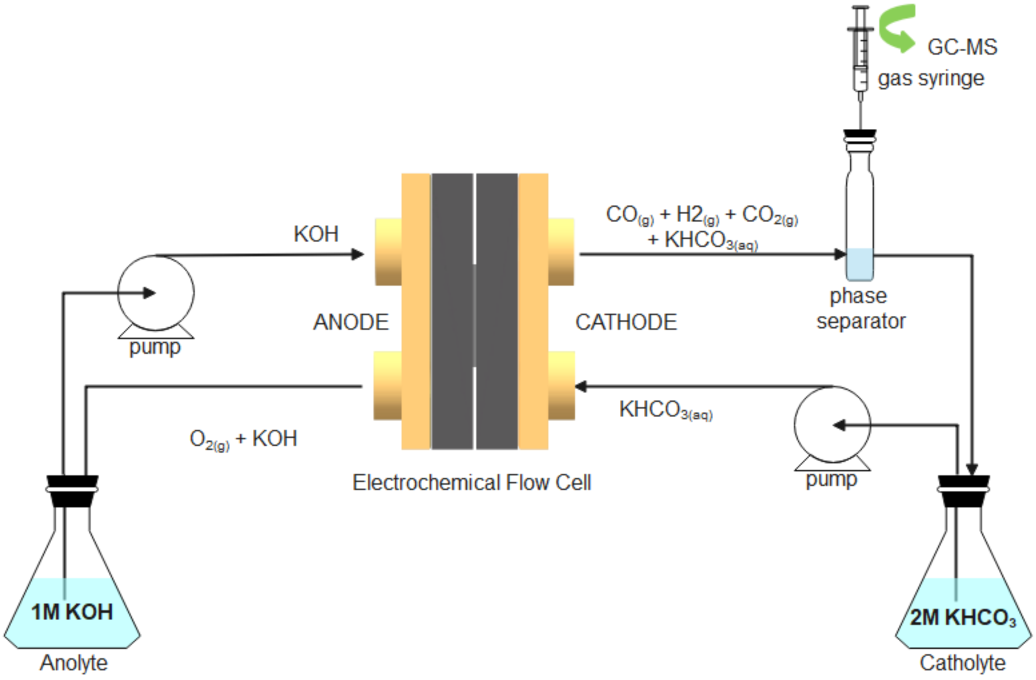


**Figure S1.** Schematic of $CO_2$ER system with bicarbonate feed used for experiments.

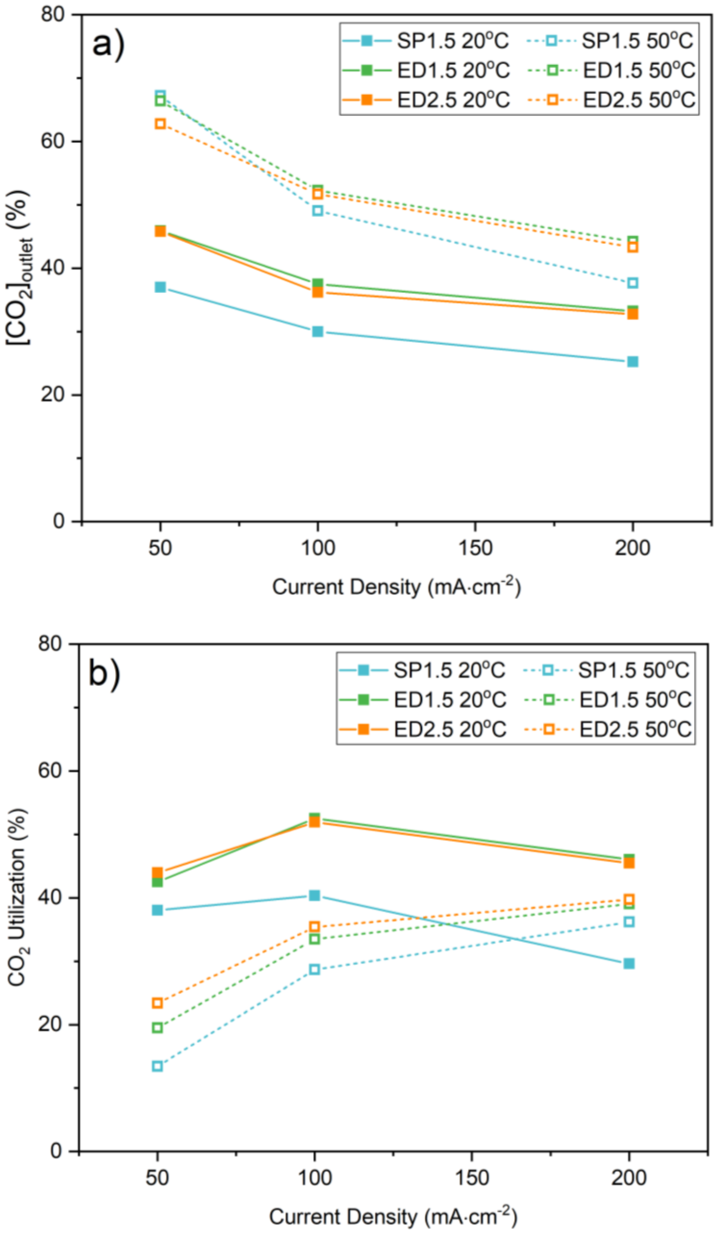


**Figure S2.** (**a**) Concentration of $CO_2$ at the outlet and (**b**) $CO_2$ Utilization for the 3 cathodes tested at 20 °C and 50 °C.

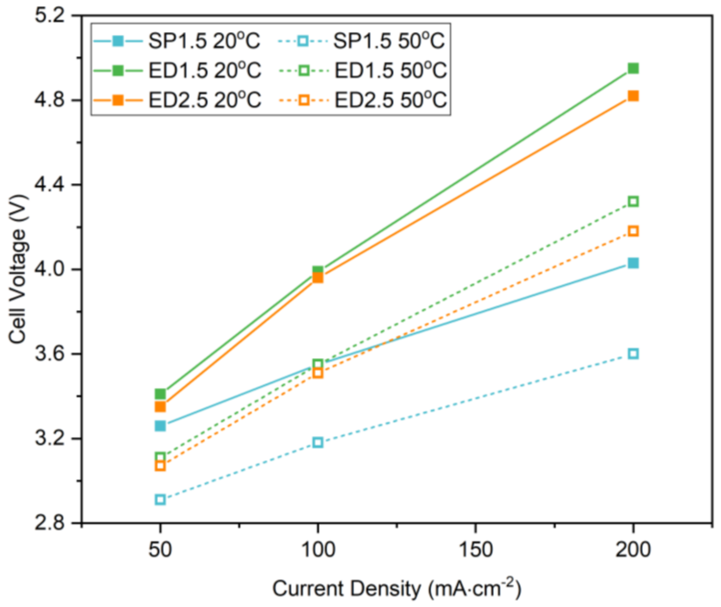


**Figure S3.** Cell voltage for the 3 cathodes tested at 20 °C and 50 °C.

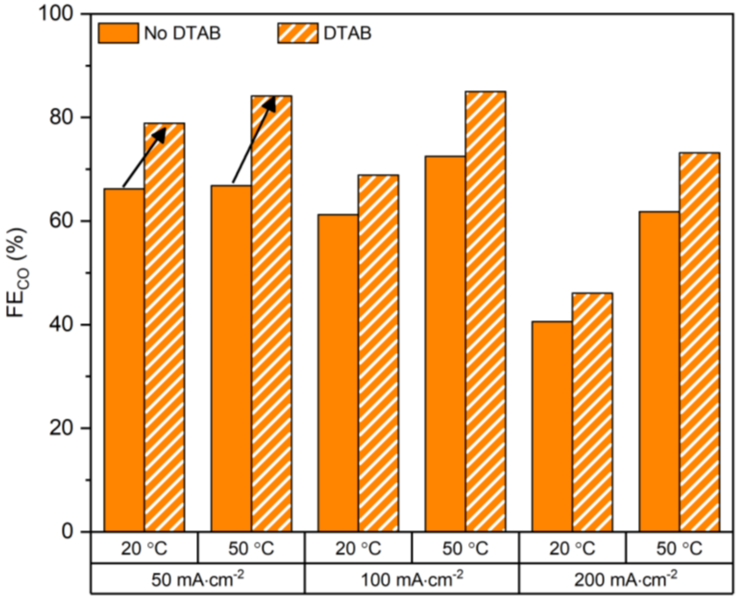


**Figure S4.** Faradaic efficiencies towards CO of system with ED2.5 cathode and catholyte with and without DTAB.

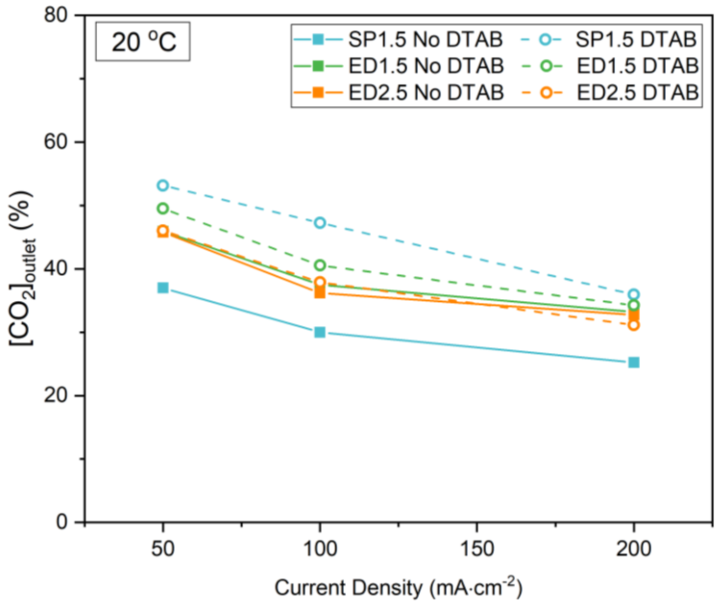


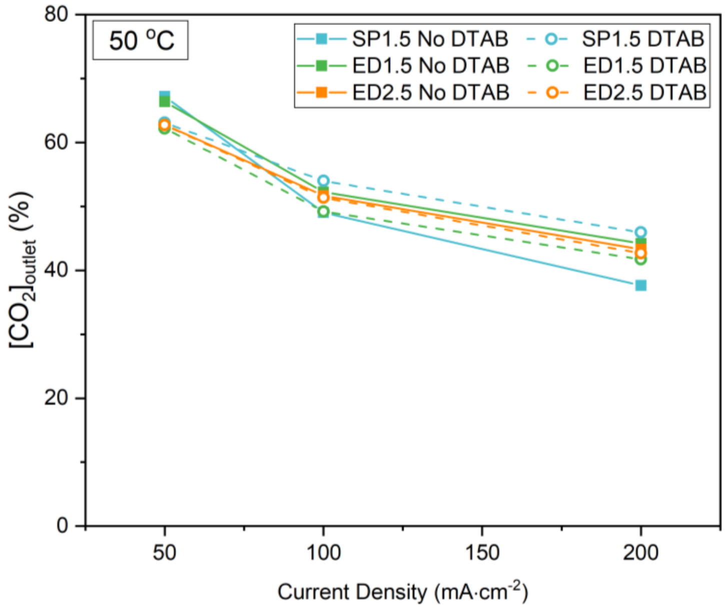


**Figure S5.** Concentration of $CO_2$ at the outlet for the 3 cathodes tested in system with catholyte with and without DTAB (**a**) 20 °C and (**b**) 50 °C.
